# Supplementary material for: Transcriptional responses of cancer cells to heat shock-inducing stimuli involve amplification of robust HSF1 binding
Source: Nat Commun. 2023 Nov 16;14:7420. doi: 10.1038/s41467-023-43157-7 (PMC10654513; doi:10.1038/s41467-023-43157-7)
Supplement: Supplementary file 1 — Supplementary Information [file 41467_2023_43157_MOESM1_ESM.pdf]

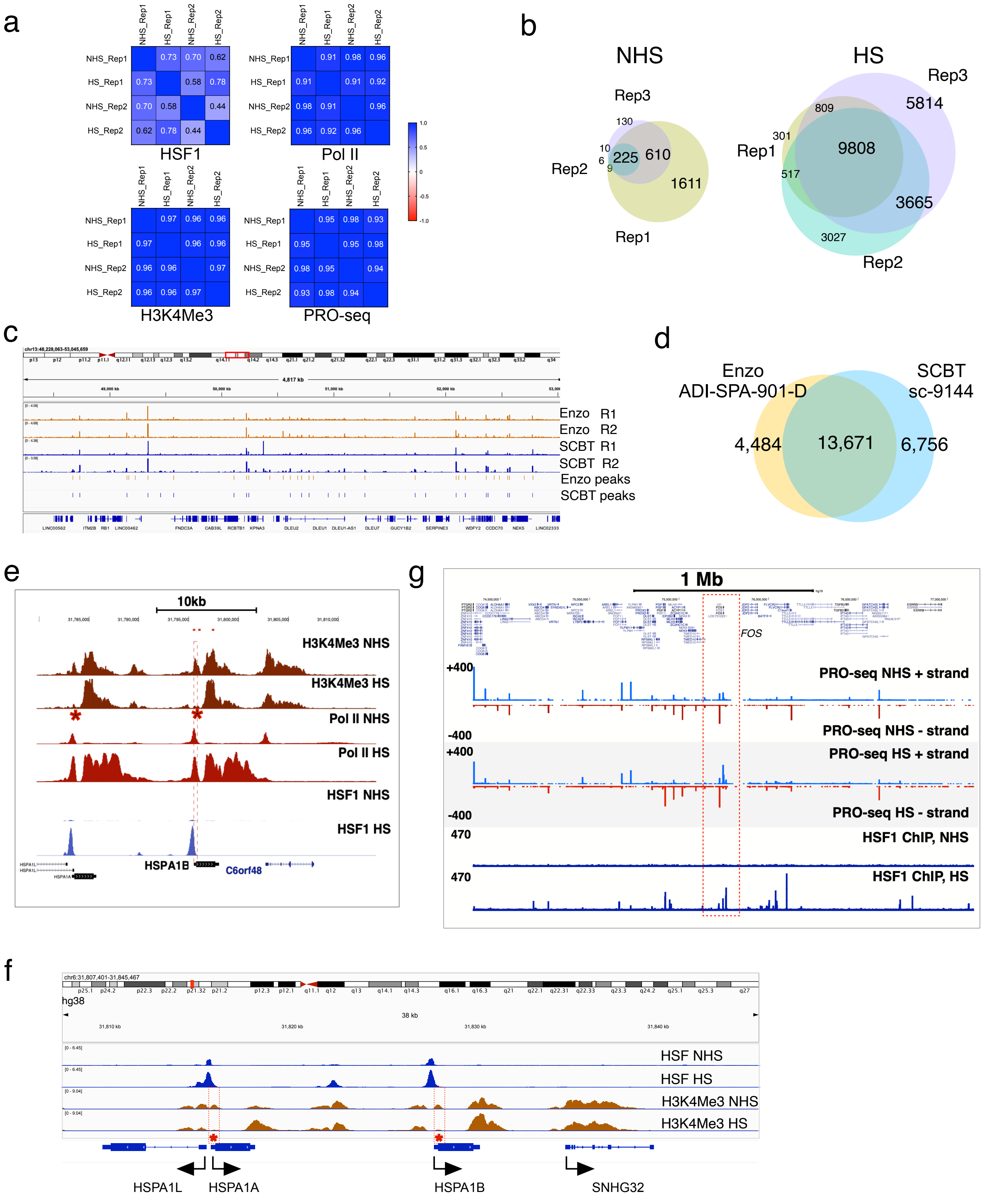

Supplementary Fig. 1

Supplementary Figure 1. a. Correlation among MCF7 datasets. Spearman correlation based on counts within peak regions for HSF1 ChIP-seq, promoter regions (+/- 1kb) for other ChIP-seq datasets, and gene body counts for PRO-seq datasets. **b.** Numbers of HSF1 peaks per replicate. Shown are overlaps for NHS (left) and HS (right) numbers of peaks for MCF7 cells. Rep 1 and Rep 2 were used for subsequent analyses unless indicated otherwise. **c.** Comparison of anti-HSF1 antibodies. IGV browser ChIP-seq tracks with anti-HSF1 Enzo (orange, used throughout this work) and polyclonal SCBT antibody (blue) showing a randomly selected ~5mB region of the genome, for each replicate. The polyclonal antibody is no longer available from any supplier. **d.** Venn diagram for MCF7 ChIP-seq peaks between the two anti-HSF1 antibodies. **e.** IGV browser shot for ChIP-seq data from this work, showing hg19-based *HPA1B* gene region with reduced H3K4Me3 signal at the gene start region in HS (left) (indicated with an asterisk) and control genomic region (right). **f.** IGV browser shot for human embryonic stem cells using pre-processed data (1) showing *HPA1B* gene region, with coordinates in this panel based on hg38 genome assembly. **g.** UCSC browser track showing PRO-seq and HSF1 ChIP-seq tracks for a ~3MB genomic region around a moderately HS-activated gene in MCF7 cells (*FOS*).

a

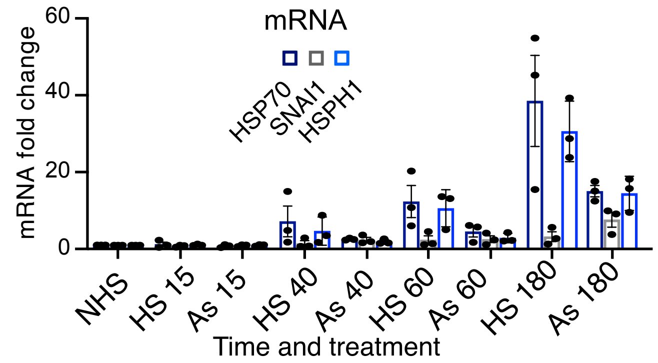

b

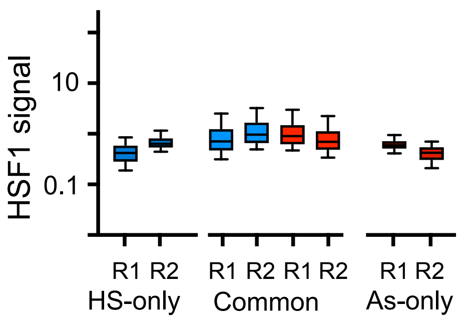

c

Common HSF1 peaks

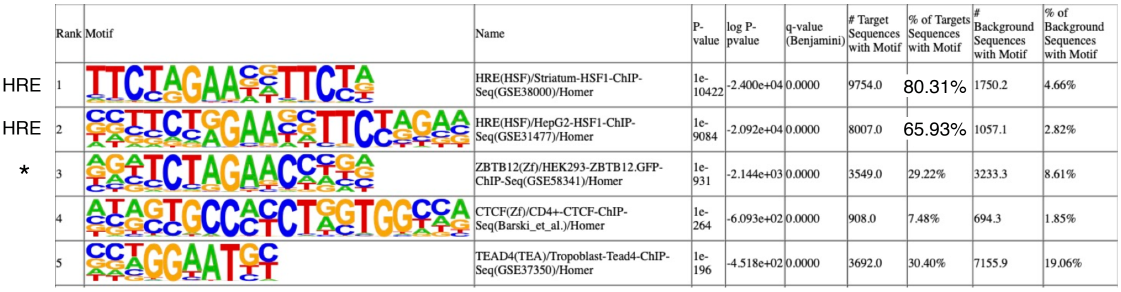

	Rank	Motif	Name	P-value	log P-pvalue	q-value (Benjamini)	# Target Sequences with Motif	% of Targets Sequences with Motif	# Background Sequences with Motif	% of Background Sequences with Motif
HRE	1		HRE(HSF)/Striatum-HSF1-ChIP-Seq(GSE38000)/Homer	1e-10422	-2.400e+04	0.0000	9754.0	80.31%	1750.2	4.66%
HRE	2		HRE(HSF)/HepG2-HSF1-ChIP-Seq(GSE31477)/Homer	1e-9084	-2.092e+04	0.0000	8007.0	65.93%	1057.1	2.82%
*	3		ZBTB12(Zf)/HEK293-ZBTB12.GFP-ChIP-Seq(GSE58341)/Homer	1e-931	-2.144e+03	0.0000	3549.0	29.22%	3233.3	8.61%
	4		CTCF(Zf)/CD4+-CTCF-ChIP-Seq(Barski_et_al.)/Homer	1e-264	-6.093e+02	0.0000	908.0	7.48%	694.3	1.85%
	5		TEAD4(TEA)/Tropoblast-Tead4-ChIP-Seq(GSE37350)/Homer	1e-196	-4.518e+02	0.0000	3692.0	30.40%	7155.9	19.06%

HS-only HSF1 peaks

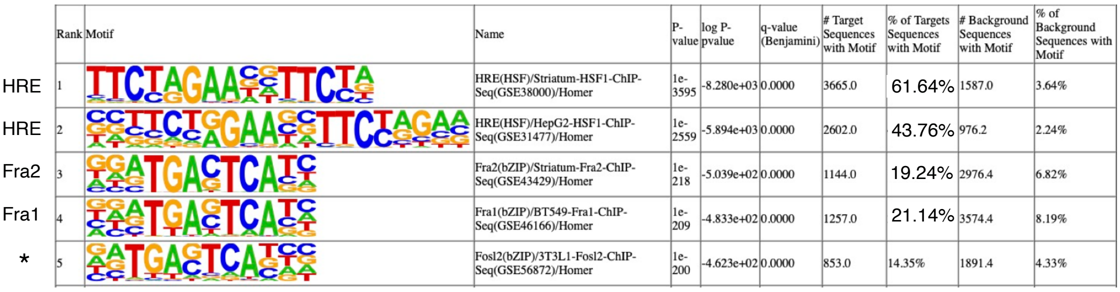

	Rank	Motif	Name	P-value	log P-pvalue	q-value (Benjamini)	# Target Sequences with Motif	% of Targets Sequences with Motif	# Background Sequences with Motif	% of Background Sequences with Motif
HRE	1		HRE(HSF)/Striatum-HSF1-ChIP-Seq(GSE38000)/Homer	1e-3595	-8.280e+03	0.0000	3665.0	61.64%	1587.0	3.64%
HRE	2		HRE(HSF)/HepG2-HSF1-ChIP-Seq(GSE31477)/Homer	1e-2559	-5.894e+03	0.0000	2602.0	43.76%	976.2	2.24%
Fra2	3		Fra2(bZIP)/Striatum-Fra2-ChIP-Seq(GSE43429)/Homer	1e-218	-5.039e+02	0.0000	1144.0	19.24%	2976.4	6.82%
Fra1	4		Fra1(bZIP)/BT549-Fra1-ChIP-Seq(GSE46166)/Homer	1e-209	-4.833e+02	0.0000	1257.0	21.14%	3574.4	8.19%
*	5		Fosl2(bZIP)/3T3L1-Fosl2-ChIP-Seq(GSE56872)/Homer	1e-200	-4.623e+02	0.0000	853.0	14.35%	1891.4	4.33%

As-only HSF1 peaks

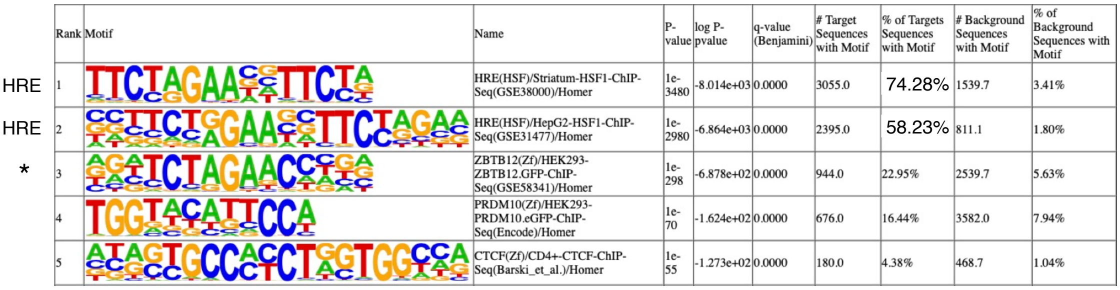

	Rank	Motif	Name	P-value	log P-pvalue	q-value (Benjamini)	# Target Sequences with Motif	% of Targets Sequences with Motif	# Background Sequences with Motif	% of Background Sequences with Motif
HRE	1		HRE(HSF)/Striatum-HSF1-ChIP-Seq(GSE38000)/Homer	1e-3480	-8.014e+03	0.0000	3055.0	74.28%	1539.7	3.41%
HRE	2		HRE(HSF)/HepG2-HSF1-ChIP-Seq(GSE31477)/Homer	1e-2980	-6.864e+03	0.0000	2395.0	58.23%	811.1	1.80%
*	3		ZBTB12(Zf)/HEK293-ZBTB12.GFP-ChIP-Seq(GSE58341)/Homer	1e-298	-6.878e+02	0.0000	944.0	22.95%	2539.7	5.63%
	4		PRDM10(Zf)/HEK293-PRDM10.eGFP-ChIP-Seq(Encode)/Homer	1e-70	-1.624e+02	0.0000	676.0	16.44%	3582.0	7.94%
	5		CTCF(Zf)/CD4+-CTCF-ChIP-Seq(Barski_et_al.)/Homer	1e-55	-1.273e+02	0.0000	180.0	4.38%	468.7	1.04%

d

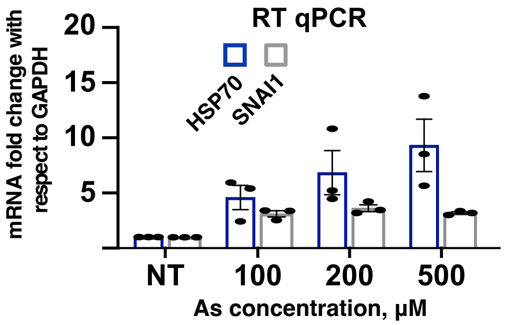

e

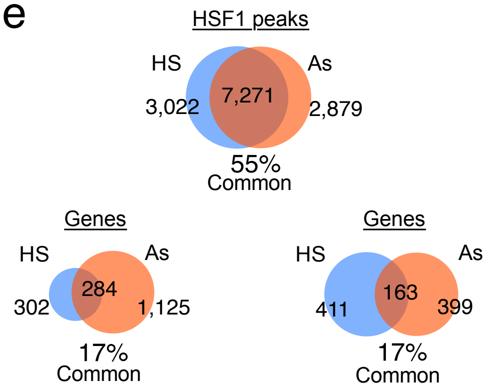

Supplementary Fig. 2

Supplementary Figure 2. a. RT-qPCR showing titration of As treatment timing. HS treatment was performed in parallel for the same time points and is shown next to As datapoints done under 500 micromolar sodium meta-arsenate. Each experiment is based on three independent biological replicates, shown with dots. The boxplot shows the mean with SEM. **b.** HSF1 signal for MCF7 cells for all peaks that are common for HS and As treatments or exclusive to each treatment. HS signal is shown in blue and As in red. Boxplots show medians, top and bottom quartiles, with whiskers showing 10-90% interval.The number of peaks in each category is shown below the boxplots. **c.** Enrichment of transcription factor motifs for HSF1 peaks between HS and As treatments. The top five transcription factor motif hits sorted by the p-value are shown as output of homer known motifs. Highlighted are HRE motifs as well as putative motifs for AP-1 family factors Fra1 and Fra2, alongside the percentage of sequences with the corresponding motifs. Other motifs shown are not highlighted as they either have too low representation (<20% of sequences with motif), higher p-value, or a sequence that resembles that of HRE. Asterisks indicate motifs assigned to other factors, but that clearly resemble the highlighted motif sequences. **d.** RT-qPCR showing titration of As concentration in MCF7 cells, with timing fixed at 60 minutes. Each experiment is based on three independent biological replicates, shown with dots. The boxplot shows the mean with SEM. **e.** Overlaps between HS and As treatments with increased stringency. *Top.* Venn diagram showing the overlap between HSF1 peaks observed in HS and As treatments in MCF7 cells showing only the identified in both replicates. *Bottom left.* The overlap between activated genes as detected by PRO-seq using a minimum absolute gene expression (0.001 reads per million per kilobase) and a minimum fold change in at activated genes as 1.4. *Bottom right.* Activated genes as on the left, but with the same number of genes in HS and As conditions after sorting the activated genes based on fold-change. The percentage of commonly activated genes among all genes is shown underneath.

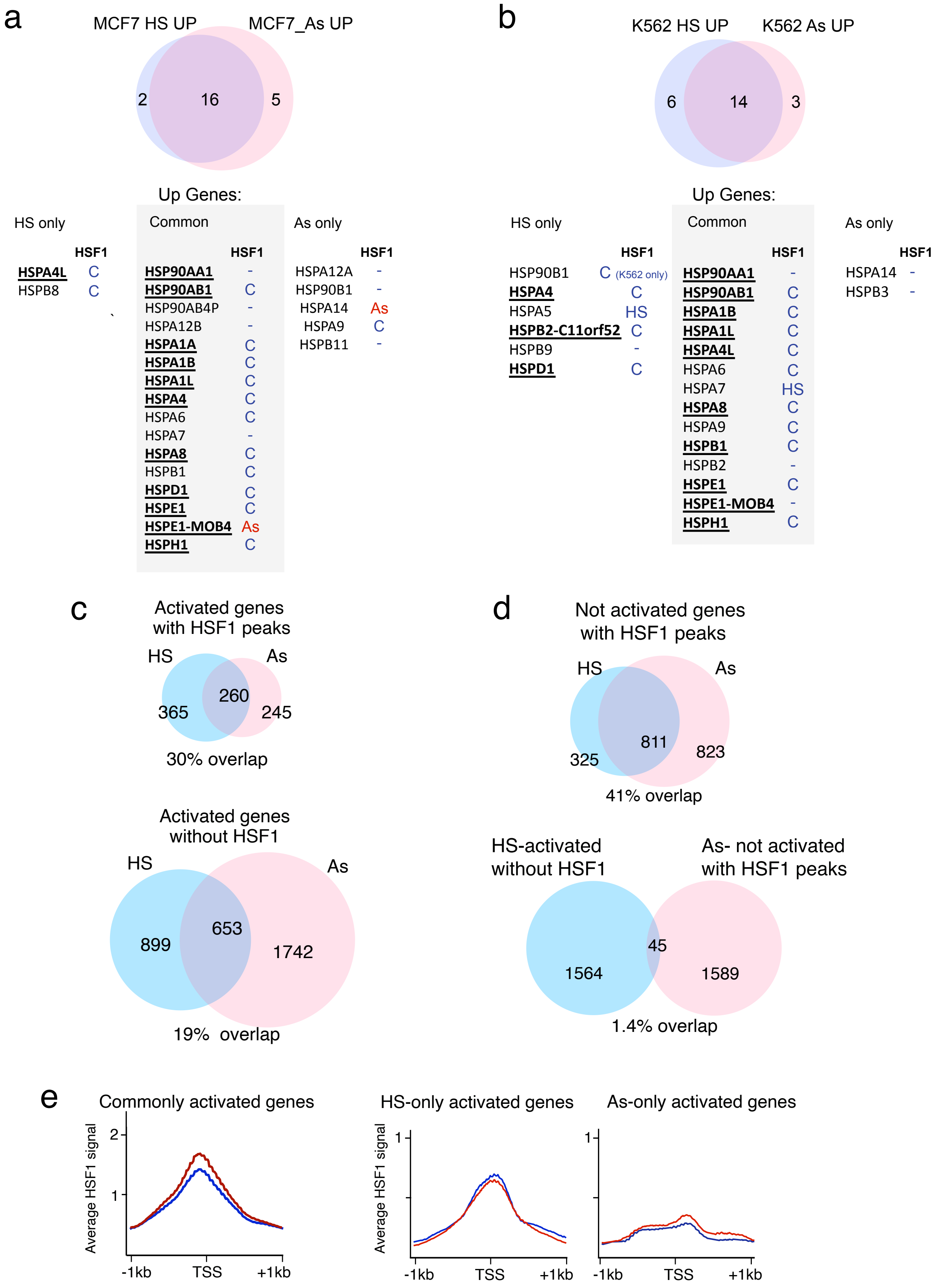

Supplementary Fig. 3

Supplementary Figure 3. a. HSF1 binding and transcriptional activation of genes annotated as HSP in MCF7 cells. *Top.* Overlap between HSP genes activated in HS and As. *Bottom.* Gene names shown for each slice of the Venn diagram. HSF1 binding status is shown next to each gene, with condition indicated (HS (HS-only); As (As-only), "c" (common, present both in HS and As); "-" no HSF1). Genes activated in K562 cells as defined previously (2) are shown in bold font. **b.** The same as in (a), but for K562 datasets. **c.** Overlap in genes activated in HS and As treatments with HSF1 peaks at promoters (top) and without HSF1 peaks (bottom). **d.** Overlap in genes not activated in HS and As treatments with HSF1 peaks at promoters (top). Negative control overlap between HS-activated genes without HSF1 and As non-activated genes with HSF1 (bottom). Percentage overlaps shown below indicate a fraction of overlapping genes against all genes in the Venn diagram. **e.** HSF1 binding between HS and As treatments. Metaplot of HSF1 ChIP- seq profiles for promoters whose genes are activated in both treatments based on PRO-seq (*left*) or bound in both treatments, but activated in only one (*right*). The Y-axis scale is similar for all plots.

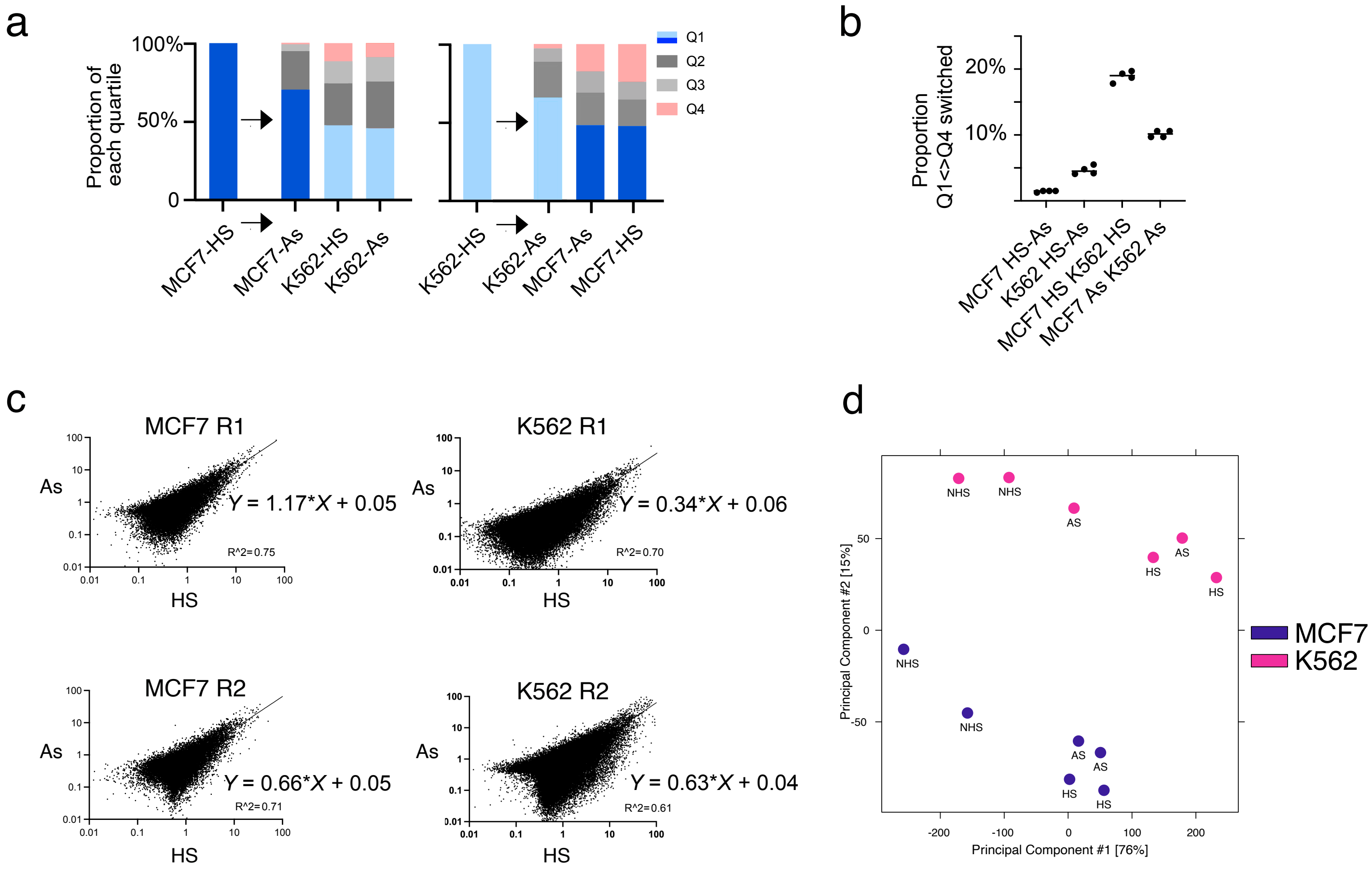

Supplementary Fig. 4

Supplementary Figure 4. a. Bar graph showing the changes of HSF1 peaks between quartiles sorted by signal within peak regions. Distribution of Quartile 1 (highest signal, Q1) peaks for MCF7 HS (left, dark blue) and K562 HS (right, light blue) are shown across each quartile for conditions indicated below the graph. **b.** Percentage of HSF1 peaks switching between the extreme quartiles (defined as a sum of peaks switching between Q1->Q4 plus Q4->Q1) at the indicated conditions, for each pair of replicates (4 datapoints total per condition). **c.** Scatterplots of normalized HSF1 peak counts between HS and As conditions for the indicated replicates. Simple linear regression is shown for each plot. **d.** Principal Component Analysis for indicated samples (per replicate) using a subset of HSF1 peaks that are common among all conditions (MCF7 HS, As and K562 HS, As).

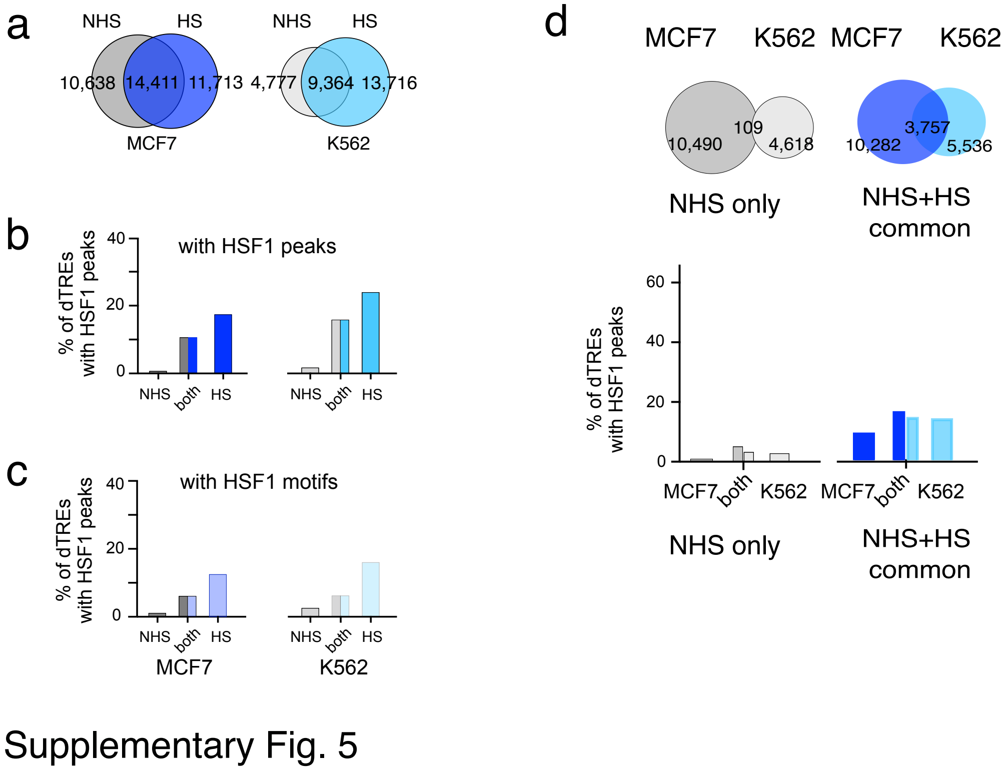

Supplementary Figure 5. a. Overlap between dTREs called in NHS and HS conditions for each cell line. **b.** Percentage of dTREs with HSF1 peaks in NHS and HS treatments for each cell line including NHS-only, common, and HS-only dTREs. **c.** Percentage of dTREs with HRE sequence motifs for each cell line in NHS and HS treatments for the same dTRE groups. **d.** (*Top*) Overlap between NHS only and NHS and HS common dTREs between MCF7 and K562 cell lines. (*Bottom*) The percentage of dTREs with HSF1 ChIP-seq peaks NHS only and NHS+HS common dTREs. Source data are provided as a Source Data file.

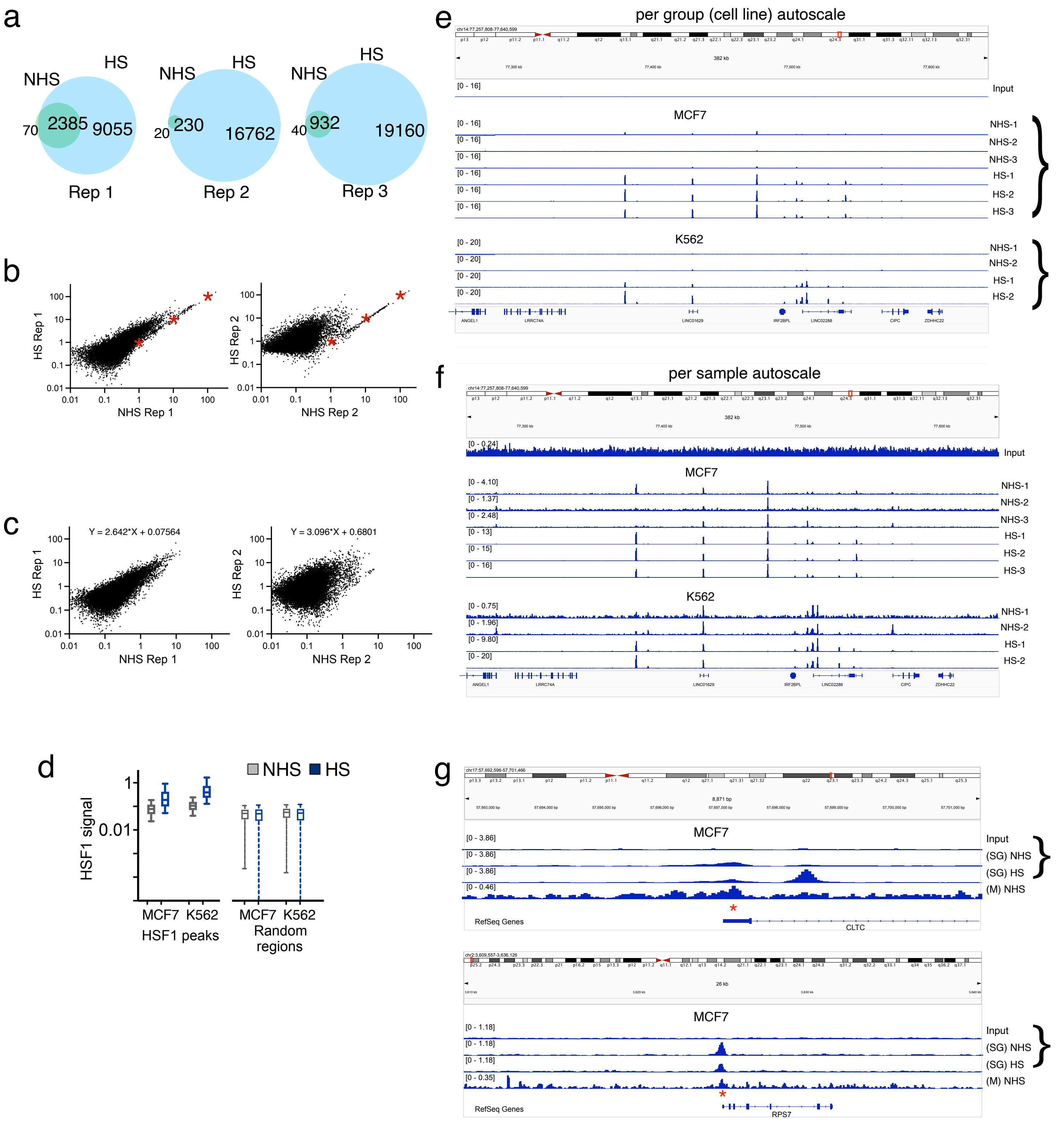

Supplementary Fig. 6

Supplementary Figure 6. a. Overlap between peaks called at NHS and HS conditions for each individual MCF7 replicate. **b.** Scatterplots of HSF1 signal NHS versus HS samples for the indicated replicates processed without removal of blacklisted regions (done only for this graph). Red asterisks indicate the slope of 1 after normalizing using counts per million (CPM). **c.** Scatterplots of the same samples as in (b), except blacklisted regions were removed (the default processing for all peaks). **d.** NHS signal compared to the random background. The box plot shows HS-only peaks in NHS and HS cells for each cell line as indicated (15,698 for MCF7 and 30,360 for K562) and signal from locations selected using *bedtools random* in the same datasets (29,036 for each cell line). The box plots show medians with top and bottom quartiles, and error bars indicating 10-90% intervals. **e.** IGV browser shot of NHS and HS datasets used in this work, for each replicate. The tracks are group-scaled within each cell line to emphasize NHS versus HS differences, with groups shown with curved brackets. **f.** The same IGV browser shot as in (e), except tracks are autoscaled within individual datasets to emphasize the locations of enrichment in NHS samples. **g.** IGV browser shot of NHS-only peak regions (shown with asterisks) for NHS datasets from this work (SG) and previously published NHS MCF7 datasets (M) (3). The current (SG) datasets are group-scaled as indicated by brackets.

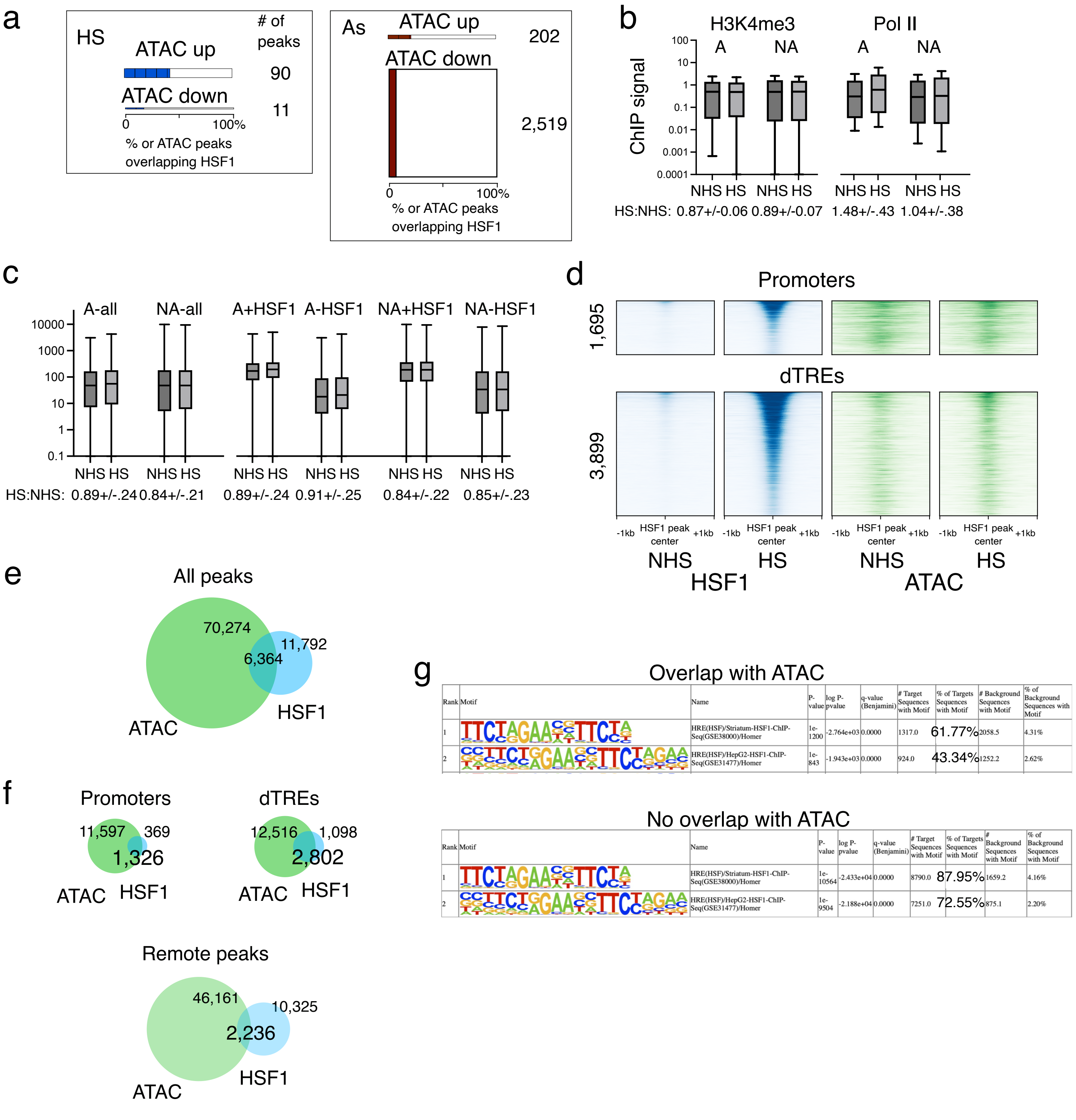

Supplementary Fig. 7

Supplementary Figure 7. a. Enrichment of HSF1 binding among up and downregulated ATAC regions. Horizontal bars show the numbers of up- and down-regulated ATAC peaks for HS (*left*) and As (*right*) treatments in MCF7 cells. The vertical heights of the bars are scaled to the number of peaks in each category (the numbers of peaks indicated on the right). The percentage of HSF1 peaks in each ATAC peak category is shown in the x-axis in blue (HS) and red (As). **b.** Signal for H3K4me3 and Pol II ChIP-seq experiments from our data at promoters of activated (A) or non-activated (NA) genes in NHS and HS conditions. Numbers indicate the ratios of means of ChIP-seq signal from activated:non-activated genes, based on a range from two biological replicates. Boxplots show medians with top and bottom quartiles, with whiskers indicating 10-90th percentiles. **c.** Boxplots of ATAC signal counts for all genes as in (b) and genes broken down into those containing and not containing HSF1 at promoters (+/- 1kb from the TSS). Boxplots show medians with top and bottom quartiles, and whiskers show the data range min to max. Numbers underneath show the number of genes in each NHS vs HS comparison and NHS:HS signal ratios between average signal and range between two independent biological replicates. **e** Overlap between all ATAC and HSF1 HS peaks. **f.** Overlap between ATAC and HSF1 peaks mapping to indicated functional elements. The numbers of HSF1 peaks overlapping with ATAC peaks are shown in larger, underlined font. **d.** HSF1 and ATAC signal in functional genomic elements. HSF1 is binding to already open genomic elements. NHS and HS datapoints are shown for MCF7 cells for elements that overlap with HSF1 peaks including promoters and dTREs. The numbers of elements in each category are shown on the left of each heatmap. **g.** Abundance of HRE sequence motifs between distant open and closed HSF1 peaks. HSF1 peaks outside of promoters, dTREs, and annotated enhancers were separated into those that do and those that do not overlap with ATAC peaks, followed by homer known motif identification. HSF1 peaks that are more closed based on the absence of ATAC peaks show higher % of targets with HRE elements than those that are more open, that is, overlap with ATAC peaks.

SUPPLEMENTARY REFERENCES.

1. Lyu, X., Rowley, M. J. & Corces, V. G. Architectural Proteins and Pluripotency Factors Cooperate to Orchestrate the Transcriptional Response of hESCs to Temperature Stress. *Mol Cell* **71**, 940-955.e7 (2018).
2. Vihervaara, A. et al. Transcriptional response to stress in the dynamic chromatin environment of cycling and mitotic cells. *Proc Natl Acad Sci U S A* **110**, E3388-97 (2013).
3. Mendillo, M. L. et al. HSF1 drives a transcriptional program distinct from heat shock to support highly malignant human cancers. *Cell* **150**, 549-562 (2012).
