# Supplementary material for: Transcriptional responses of cancer cells to heat shock-inducing stimuli involve amplification of robust HSF1 binding
Source: Nat Commun. 2023 Nov 16;14:7420. doi: 10.1038/s41467-023-43157-7 (PMC10654513; doi:10.1038/s41467-023-43157-7)
Supplement: Supplementary file 3 — Description of Additional Supplementary Files [file 41467_2023_43157_MOESM3_ESM.pdf]

Description of additional supplementary files

File name: Supplementary Data 1

Description: Omics datasets generated in this study including sequencing depth and alignment statistics.

File name: Supplementary Data 2

Description: Coordinates of HSF1 peaks for each condition including chromosome, peak start, peak end, based on hg19 genome assembly.

File name: Supplementary Date 3

Description: Promoters of upregulated genes for each condition, including chromosome, interval start, interval end, gene symbol, strand. Intervals are defined as transcription start site +/-1000 nucleotides.

File name: Supplementary Data 4

Description: Panther GO Ontology over-representation test for upregulated genes in MCF7 cells that are commonly activated in HS and As, unique to HS and unique to As.

File name: Supplementary Data 5

Description: Panther GO Ontology over-representation test for HS upregulated genes in MCF7 and K562 cells including common between MCF7 cells, unique to MCF7 and unique to K562 cells.

File name: Supplementary Data 6

Description: Coordinates of dTREs identified in this study in the cell line and condition indicated, including chromosome, dTRE start, dTRE end.

File name: Supplementary Date 7

Description: Promoters associated with basal (NHS-only) peaks in MCF7 cells, including coordinates and names of the corresponding genes as well as GO Gene Ontology PANTHER overrepresentation test.

File name: Supplementary Data 8

Description: Primers used in this study. All primers are from IDTDNA. Primers for qPCR were synthesized in 25 nmol scale without additional purification. Linkers for PRO-seq were synthesized in 100 nmol scale with HPLC purification.
