# Supplementary material for: Transcriptional responses of cancer cells to heat shock-inducing stimuli involve amplification of robust HSF1 binding
Source: Nat Commun. 2023 Nov 16;14:7420. doi: 10.1038/s41467-023-43157-7 (PMC10654513; doi:10.1038/s41467-023-43157-7)
Supplement: Supplementary file 12 — Reporting Summary [file 41467_2023_43157_MOESM12_ESM.pdf]

nature portfolio

Corresponding author(s): Sergei Nechaev
Last updated by author(s): Jan 27, 2023

Reporting Summary

Nature Portfolio wishes to improve the reproducibility of the work that we publish. This form provides structure for consistency and transparency in reporting. For further information on Nature Portfolio policies, see our Editorial Policies and the Editorial Policy Checklist.

Statistics

For all statistical analyses, confirm that the following items are present in the figure legend, table legend, main text, or Methods section.

n/a	Confirmed	
☐	☒	The exact sample size (n) for each experimental group/condition, given as a discrete number and unit of measurement
☐	☒	A statement on whether measurements were taken from distinct samples or whether the same sample was measured repeatedly
☐	☒	The statistical test(s) used AND whether they are one- or two-sided<br>*Only common tests should be described solely by name; describe more complex techniques in the Methods section.*
☒	☐	A description of all covariates tested
☒	☐	A description of any assumptions or corrections, such as tests of normality and adjustment for multiple comparisons
☐	☒	A full description of the statistical parameters including central tendency (e.g. means) or other basic estimates (e.g. regression coefficient) AND variation (e.g. standard deviation) or associated estimates of uncertainty (e.g. confidence intervals)
☐	☒	For null hypothesis testing, the test statistic (e.g. F, t, r) with confidence intervals, effect sizes, degrees of freedom and P value noted<br>*Give P values as exact values whenever suitable.*
☒	☐	For Bayesian analysis, information on the choice of priors and Markov chain Monte Carlo settings
☒	☐	For hierarchical and complex designs, identification of the appropriate level for tests and full reporting of outcomes
☒	☐	Estimates of effect sizes (e.g. Cohen's d, Pearson's r), indicating how they were calculated

Our web collection on statistics for biologists contains articles on many of the points above.

Software and code

Policy information about availability of computer code

Data collection	No software was used for data collection.
Data analysis	Open-source code was used including trimmomatic 0.39, hisat2 2.1.0, samtools 1.10, bedtools 2.29.0, bamcoverage 3.3, Picard MarkDuplicates 2.20.2, macs2 2.2.6, DiffBind 3.0.11, R venneuler package 1.1.3, HOMER 4.11, DeSeq2 1.30, deepTools 3.3.

For manuscripts utilizing custom algorithms or software that are central to the research but not yet described in published literature, software must be made available to editors and reviewers. We strongly encourage code deposition in a community repository (e.g. GitHub). See the Nature Portfolio guidelines for submitting code & software for further information.

Data

Policy information about availability of data

All manuscripts must include a data availability statement. This statement should provide the following information, where applicable:

- Accession codes, unique identifiers, or web links for publicly available datasets
- A description of any restrictions on data availability
- For clinical datasets or third party data, please ensure that the statement adheres to our policy

The sequencing data generated in this study have been deposited in the Gene Expression Omnibus (GEO) database under accession code GSE209687 [https://www.ncbi.nlm.nih.gov/geo/query/acc.cgi?acc=GSE209687]. Sequencing data processed in this study and original non-sequencing data are provided in the Supplementary Information and Source Data file. Source data are provided with this paper. All data used in this study are based on hg19 human genome assembly.

Public ChIP-seq datasets analyzed during the current study are under GEO accession codes GSE85158 [https://www.ncbi.nlm.nih.gov/geo/query/acc.cgi?acc=GSE85158], GSE105028 [https://www.ncbi.nlm.nih.gov/geo/query/acc.cgi?acc=GSE105028], GSE38912 [https://www.ncbi.nlm.nih.gov/geo/query/acc.cgi?acc=GSE38912], and GSE43579 [https://www.ncbi.nlm.nih.gov/geo/query/acc.cgi?acc=GSM2367735].

Human research participants

Policy information about studies involving human research participants and Sex and Gender in Research.

Reporting on sex and gender	N/A
Population characteristics	N/A
Recruitment	N/A
Ethics oversight	N/A

Note that full information on the approval of the study protocol must also be provided in the manuscript.

Field-specific reporting

Please select the one below that is the best fit for your research. If you are not sure, read the appropriate sections before making your selection.

☒ Life sciences ☐ Behavioural & social sciences ☐ Ecological, evolutionary & environmental sciences

For a reference copy of the document with all sections, see nature.com/documents/nr-reporting-summary-flat.pdf

Life sciences study design

All studies must disclose on these points even when the disclosure is negative.

Sample size	No test was used to define the sample size. ChIP-seq data were collected in a minimum of 2 independent biological replicates, and validated by qPCR prior to sequencing. Studies involving qPCR were done in 3+ independent biological replicates.
Data exclusions	PRO-seq datasets generated from the same cell batches as ChIP-seq data were excluded from further analyses due to technical failure of PRO-seq library preparation, but were still validated by PCR for appropriate gene expression response. PRO-seq data were re-generated from the same passage number of the original commercial cell stock. No data were excluded in studies involving qPCR.
Replication	Genomics datasets from independently obtained biological duplicates. Biological replicates of the same treatment were separated in time by several months.
Randomization	Randomization is not relevant to our study as it relates on pairwise comparisons between datapoints.
Blinding	Data collection itself was not blinded. However, data analyses were done with numerical sample identifiers without knowing treatment identity.

Reporting for specific materials, systems and methods

We require information from authors about some types of materials, experimental systems and methods used in many studies. Here, indicate whether each material, system or method listed is relevant to your study. If you are not sure if a list item applies to your research, read the appropriate section before selecting a response.

Materials & experimental systems

n/a	Involved in the study	
☐	☒	Antibodies
☐	☒	Eukaryotic cell lines
☒	☐	Palaeontology and archaeology
☒	☐	Animals and other organisms
☒	☐	Clinical data
☒	☐	Dual use research of concern

Methods

n/a	Involved in the study	
☐	☒	ChIP-seq
☒	☐	Flow cytometry
☒	☐	MRI-based neuroimaging

Antibodies

Antibodies used	anti-HSF1: HSF1 (H-311) sc 9144 (lot # h0213) was used for initial experiments. This rabbit polyclonal antibody was permanently discontinued by the manufacturer in ~2016.<br>anti-HSF1: Enzo ADI-SPA-901-F, lot# 07311706<br>anti-phospho HSF1: Enzo ADI-SPA-902-D, lot# 09061832<br>anti Pol II Rpb1 NTD: (D8L4Y) (CST #14958), Lot 1<br>anti histone H3K4Me3: Abcam ab8580, Lot GR3205191-1<br>Secondary antibody for western blot was GE #NA934 (currently Cytiva under the same catalog number).
Validation	Each antibody used here has been previously validated. The histone and Pol II antibodies are most frequently used elsewhere, including for ChIP-seq.<br>https://www.cellsignal.com/products/primary-antibodies/rpb1-ntd-d8l4y-rabbit-mab/14958<br>https://www.abcam.com/histone-h3-tri-methyl-k4-antibody-chip-grade-ab8580.html<br>In our hands, these antibodies produce expected ChIP profiles, display appropriate subcellular localization, and appropriate sizes on Western blots.<br>The pan HSF1 antibodies have been previously used, including for ChIP-seq<br>https://www.enzolifesciences.com/ADI-SPA-901/hsf1-polyclonal-antibody/<br>In our hands, the Enzo antibody displays appropriate sizing on Western Blotting and appropriate, stimulus-dependent binding to its DNA targets.<br>The phospho-specific antibody has been used only for Western https://www.enzolifesciences.com/ADI-SPA-902/pser326-hsf1-polyclonal-antibody/<br>In our hands, ADI-SPA-902 antibody displays appropriate Western band size and dependence of signal on cell stimulation, as appropriate for HSF1 activation upon stress.

Eukaryotic cell lines

Policy information about cell lines and Sex and Gender in Research

Cell line source(s)	The MCF-7 and K562 cell lines used here were purchased directly from ATCC and used within the first 15 passages
Authentication	Cells were verified morphologically and for expression levels of marker genes such as SNAI1. In addition, omics datasets obtained from these cell lines were verified by examining genome browser tracks against positions of known copy number variations from Cancer Cell Line Encyclopedia:<br>Barretina,J. et al,(2012) The Cancer Cell Line Encyclopedia enables predictive modelling of anticancer drug sensitivity. Nature 483, 603-607.
Mycoplasma contamination	Cells from ATCC are guaranteed mycoplasma-free so we have not tested these specific batches that were used up in early passages. Prior tests of MCF7 and other cells, and tests since have been negative for mycoplasma.
Commonly misidentified lines<br>(See ICLAC register)	No commonly misidentified lines were used in this study.

ChIP-seq

Data deposition

☒ Confirm that both raw and final processed data have been deposited in a public database such as GEO.

☒ Confirm that you have deposited or provided access to graph files (e.g. BED files) for the called peaks.

Data access links<br>*May remain private before publication.*	https://www.ncbi.nlm.nih.gov/geo/query/acc.cgi?acc=GSE209687
Files in database submission	Raw files: s18_133_004_1.fq.gz s18_133_004_2.fq.gz<br>s18_133_005_1.fq.gz s18_133_005_2.fq.gz<br>s18_133_006_1.fq.gz s18_133_006_2.fq.gz<br>s18_088_001_1.fq.gz s18_088_001_2.fq.gz<br>s18_088_002_1.fq.gz s18_088_002_2.fq.gz<br>s18_088_003_1.fq.gz s18_088_003_2.fq.gz<br>18-068-002_S2_L001_R1_001.fastq.gz 18-068-002_S2_L001_R2_001.fastq.gz<br>18-068-004_S4_L001_R1_001.fastq.gz 18-068-004_S4_L001_R2_001.fastq.gz<br>18-068-006_S2_L001_R1_001.fastq.gz 18-068-006_S2_L001_R2_001.fastq.gz<br>18-068-008_S4_L001_R1_001.fastq.gz 18-068-008_S4_L001_R2_001.fastq.gz<br>G2020_27_1_R1.fastq.gz G2020_27_1_R2.fastq.gz<br>G2020_27_2_R1.fastq.gz G2020_27_2_R2.fastq.gz<br>G2020_27_3_R1.fastq.gz G2020_27_3_R2.fastq.gz<br>G2020_27_4_R1.fastq.gz G2020_27_4_R2.fastq.gz<br>G2020_27_5_R1.fastq.gz G2020_27_5_R2.fastq.gz<br>G2020_27_6_R1.fastq.gz G2020_27_6_R2.fastq.gz<br>G2020_27_7_R1.fastq.gz G2020_27_7_R2.fastq.gz<br>G2020_27_8_R1.fastq.gz G2020_27_8_R2.fastq.gz<br>G2020_27_9_R1.fastq.gz G2020_27_9_R2.fastq.gz

G2020_27_10_R1.fastq.gz G2020_27_10_R2.fastq.gz
G2020_27_11_R1.fastq.gz G2020_27_11_R2.fastq.gz
G2020_27_12_R1.fastq.gz G2020_27_12_R2.fastq.gz
HSF1-Cont-IP-1_S1_L001_R1_001.fastq.gz HSF1-Cont-IP-1_S1_L001_R2_001.fastq.gz
HSF1-Cont-IP-2_S2_L001_R1_001.fastq.gz HSF1-Cont-IP-2_S2_L001_R2_001.fastq.gz
HSF1-HS-IP-1_S1_L001_R1_001.fastq.gz HSF1-HS-IP-1_S1_L001_R2_001.fastq.gz
HSF1-HS-IP-2_S2_L001_R1_001.fastq.gz HSF1-HS-IP-2_S2_L001_R2_001.fastq.gz
G2023_11_1_S1_L004_R1_001.fastq.gz G2023_11_1_S1_L004_R2_001.fastq.gz
G2023_11_2_S2_L004_R1_001.fastq.gz G2023_11_2_S2_L004_R2_001.fastq.gz
G2023_11_3_S3_L004_R1_001.fastq.gz G2023_11_3_S3_L004_R2_001.fastq.gz
G2023_11_4_S4_L004_R1_001.fastq.gz G2023_11_4_S4_L004_R2_001.fastq.gz

Processed files:
s18_133_004_sorted_dedup_marked.bam_dedup_norm_coverage.bw
s18_133_005_sorted_dedup_marked.bam_dedup_norm_coverage.bw
s18_133_006_sorted_dedup_marked.bam_dedup_norm_coverage.bw
18-088-001_hisat.bam_sorted.bam_coverage.bw
18-088-002_hisat.bam_sorted.bam_coverage.bw
18-088-003_hisat.bam_sorted.bam_coverage.bw
18-068-002_S2_norm_coverage.bw
18-068-004_S4_norm_coverage.bw
18-068-006_S2_norm_coverage.bw
18-068-008_S4_norm_coverage.bw
G2020-27-1_hg19.bw
G2020-27-2_hg19.bw
G2020-27-3_hg19.bw
G2020-27-4_hg19.bw
G2020-27-5_hg19.bw
G2020-27-6_hg19.bw
G2020-27-7_hg19.bw
G2020-27-8_hg19.bw
G2020-27-9_hg19.bw
G2020-27-10_hg19.bw
G2020-27-11_hg19.bw
G2020-27-12_hg19.bw
HSF1-Cont-IP-1-S1-L001_sorted.bam_dedup_norm.bw
HSF1-Cont-IP-2-S2-L001_sorted.bam_dedup_norm.bw
HSF1-HS-IP-1-S1-L001_sorted.bam_dedup_norm.bw
HSF1-HS-IP-2-S2-L001_sorted.bam_dedup_norm.bw
G2023-11-1_dedup.bw
G2023-11-2_dedup.bw
G2023-11-3_dedup.bw
G2023-11-4_dedup.bw

Peak files (where available)
G2020-27-1_hg19_peaks.narrowPeak
G2020-27-2_hg19_peaks.narrowPeak
G2020-27-3_hg19_peaks.narrowPeak
G2020-27-4_hg19_peaks.narrowPeak
G2020-27-5_hg19_peaks.narrowPeak
G2020-27-6_hg19_peaks.narrowPeak
G2020-27-7_hg19_peaks.narrowPeak
G2020-27-8_hg19_peaks.narrowPeak
G2020-27-9_hg19_peaks.narrowPeak
G2020-27-10_hg19_peaks.narrowPeak
G2020-27-11_hg19_peaks.narrowPeak
G2020-27-12_hg19_peaks.narrowPeak
HSF1-Cont-IP-1-S1-L001_sorted.bam_dedup_peaks.narrowPeak
HSF1-Cont-IP-2-S2-L001_sorted.bam_dedup_peaks.narrowPeak
HSF1-HS-IP-1-S1-L001_sorted.bam_dedup_peaks.narrowPeak
HSF1-HS-IP-2-S2-L001_sorted.bam_dedup_peaks.narrowPeak
G2023-11-1_peaks.narrowPeak
G2023-11-2_peaks.narrowPeak
G2023-11-3_peaks.narrowPeak
G2023-11-4_peaks.narrowPeak

Genome browser session
(e.g. UCSC)

https://genome.ucsc.edu/s/Nsbio/Dastidar_2023_ChIP_peer_review

Methodology

Replicates

All ChIP-seq experiments were done on a minimum of two independent biological replicates. Cell cultures were grown at different times from freshly thawed freezer stocks and subjected to treatments independently.

Sequencing depth	Sequencing depth for each experiment and the numbers of mapped reads is specified in Supplementary File 1
Antibodies	See Antibodies section above for the description of antibodies used for ChIP.
Peak calling parameters	macs2 callpeak -t <bamfile> -f BAM --outdir <outbamfiledir> -g hs -q 0.01
Data quality	Data were validated by (i) visual examination of genome browser tracks at known positive control regions comparing our data to previously published data; (ii) Stress-dependent increase of signal in stress. Because HSF1 binding is stress-dependent, the number of peaks should be higher in stress and are observed to be several-fold higher (For each treatment series: MCF7 Replicate 1: untreated, HS, As, respectively, 2455, 11435, 15435 peaks for each sample; MCF7 Replicate 2: untreated, HS, As, respectively, 250, 16992, 11008 peaks. K562 Replicate 1: untreated, HS, As, respectively, 1553, 32504, 12942 peaks. K562 Replicate 2: untreated, HS, As, respectively, 7793, 56282, 42701 peaks. Our IgG libraries generated too little DNA (validating our washing conditions) and accordingly failed at the stage of PCR amplification. In regard to input, while we did not sequence sonicated input chromatin for these series, prior sequencing to the same depth of ~40M raw reads shows fewer than 100 peaks called with these parameters. (iii) High enrichment of HRE (recognized by HSF1) sequence motifs is retained throughout peak signal intensities. (iv) remote low intensity HSF1 peaks coincide with an independently observed increase in ATAC signal at the same location, indicating that the binding of HSF1 to these sites must have occurred while in cells and not due to artifacts of cell handling and/or cross-linking.
Software	Raw sequence files were adapter-trimmed using trimmomatic and aligned against the reference genome using hisat2. Following generation of bam files, they were used for peak calling with macs2.
